# Development and internal validation of a vaginal microecology-based multivariable prediction model for persistent high-risk human papillomavirus infection: a retrospective study

**DOI:** 10.3389/fmed.2026.1851813

**Published:** 2026-07-03

**Authors:** Li He, Cui Hu, Zhongping Huang, Tao Huang, Yuting Xiao

**Affiliations:** Department of Obstetrics and Gynecology, Mianzhu City People's Hospital, Mianzhu, Sichuan, China

**Keywords:** high-risk human papillomavirus, nomogram, persistent hpv positivity, prediction model, vaginal microecology

## Abstract

**Background:**

Persistent high-risk human papillomavirus (HR-HPV) infection is a key determinant of cervical carcinogenesis, yet early identification of women at increased risk of persistence remains challenging in routine clinical practice. Vaginal microecological disturbances have been associated with HR-HPV persistence, but their integration into individualized risk prediction models has been insufficiently explored.

**Objective:**

To develop and internally validate a multivariable prediction model for persistent/recurrent HR-HPV positivity at 12 months using vaginal microecological and clinical characteristics.

**Methods:**

This retrospective study included women with baseline HR-HPV positivity, available baseline vaginal microecological examination results, and completed 12-month HR-HPV follow-up testing at a tertiary hospital gynecologic outpatient clinic between January 2019 and December 2024. A total of 1,186 women were included and randomly divided into a training cohort (*n* = 830) and an internal validation cohort (*n* = 356). Persistent/recurrent HR-HPV positivity was defined as any HR-HPV positivity at the 12-month follow-up among women who were HR-HPV-positive at baseline, regardless of whether the same HR-HPV genotype was detected at both time points. Candidate predictors were selected in the training cohort using least absolute shrinkage and selection operator (LASSO) regression with ten-fold cross-validation. Variables retained at the one-standard-error criterion (λ_1se) were entered into a multivariable logistic regression model, which was subsequently presented as a nomogram. Model discrimination, calibration, and clinical utility were evaluated using the area under the receiver operating characteristic curve (AUC), calibration curves with the Hosmer–Lemeshow goodness-of-fit test, and decision curve analysis, respectively.

**Results:**

Among the 1,186 women, 354 (29.8%) had persistent/recurrent HR-HPV positivity at 12 months. LASSO regression retained seven predictors for final model development: age, smoking status, HPV16/18 infection, vaginal pH, non-Lactobacillus-dominant microbiota, bacterial vaginosis, and moderate-to-severe local inflammation. In multivariable logistic regression, all seven variables remained independently associated with persistent/recurrent HR-HPV positivity. HPV16/18 infection showed the strongest association (OR 8.564, 95% CI 5.383–13.955, *P* < 0.001). The final model showed good apparent discrimination in the training cohort (AUC 0.892, 95% CI 0.870–0.914) and maintained acceptable discrimination in the internal validation cohort (AUC 0.821, 95% CI 0.775–0.867). Calibration was acceptable in both cohorts, with Hosmer–Lemeshow *P*-values of 0.149 and 0.296, respectively. Decision curve analysis suggested potential net clinical benefit across a range of threshold probabilities in both cohorts.

**Conclusions:**

We developed and internally validated a vaginal microecology-based multivariable prediction model for persistent/recurrent HR-HPV positivity that showed promising discrimination, acceptable calibration, and potential clinical usefulness within this single-center cohort. This internally validated model may support individualized risk stratification and help identify women who may benefit from closer follow-up; however, external validation in independent cohorts is needed before broader clinical implementation.

## Introduction

Persistent infection with high-risk human papillomavirus (HR-HPV) is widely recognized as the necessary cause of cervical cancer and its precursor lesions (1, 2). Although most HR-HPV infections are transient and are cleared spontaneously by the host immune system within 1–2 years, a subset of infections persists and may progress to cervical intraepithelial neoplasia and invasive cancer (3, 4). Therefore, identifying women at increased risk of persistent HR-HPV infection is of critical importance for optimizing risk stratification, surveillance strategies, and early intervention in cervical cancer prevention programs.

A growing body of evidence suggests that, beyond viral genotype, host and environmental factors play an essential role in determining the persistence of HR-HPV infection (5, 6). Established clinical and behavioral factors, such as age, smoking, and reproductive history, have been associated with impaired viral clearance and increased risk of persistence (7, 8). Among virological factors, infection with HPV16 and HPV18 has consistently been shown to confer a substantially higher risk of persistence and progression compared with other HR-HPV types (9). However, these factors alone do not fully explain the heterogeneity in infection outcomes, indicating that additional biological mechanisms may contribute to viral persistence.

In recent years, increasing attention has been directed toward the role of the vaginal microenvironment in HPV infection dynamics. The vaginal microbiota, particularly Lactobacillus-dominant communities, is crucial for maintaining mucosal homeostasis and protecting against pathogen colonization (10). Disruption of this ecological balance, often characterized by reduced Lactobacillus abundance, increased vaginal pH, and overgrowth of anaerobic bacteria, has been linked to a range of gynecologic conditions, including bacterial vaginosis and sexually transmitted infections (11, 12). Importantly, emerging studies have reported associations between vaginal dysbiosis and HR-HPV persistence, potentially involving local immune responses, epithelial barrier function, and viral clearance mechanisms (13, 14). Inflammatory processes within the vaginal microenvironment have also been reported to be associated with sustained HR-HPV positivity (15).

Despite these advances, the integration of vaginal microecological factors into clinically applicable risk prediction models for persistent HR-HPV infection remains limited. Most existing studies have focused on individual risk factors or associations, rather than developing comprehensive, multivariable prediction tools that can be readily implemented in routine clinical practice. Moreover, few models incorporate routinely available vaginal microecological indicators, which are widely assessed in gynecologic outpatient settings and may provide valuable, yet underutilized, predictive information.

To address these gaps, the present study aimed to develop and internally validate a multivariable prediction model for persistent HR-HPV infection at 12 months, integrating both clinical characteristics and vaginal microecological parameters. By leveraging routinely collected outpatient data and applying penalized regression techniques for robust variable selection, we sought to construct a parsimonious and clinically applicable model to facilitate individualized risk assessment. Such a model may help identify women at high risk of persistent/recurrent HR-HPV positivity and inform risk-adapted follow-up strategies in cervical cancer prevention.

## Methods

### Study design

This retrospective observational study was conducted to develop and internally validate a multivariable prediction model for persistent/recurrent high-risk human papillomavirus (HR-HPV) positivity based on vaginal microecological and clinical characteristics. Women with baseline HR-HPV positivity and complete follow-up data were identified from routine gynecologic outpatient records, and the study population was randomly divided into a training cohort for model development and an internal validation cohort for split-sample internal validation.

### Setting

The study was based on data collected from a tertiary hospital gynecologic outpatient clinic between January 2019 and December 2024. During this period, women underwent routine cervical cancer screening, HR-HPV testing, and vaginal microecological assessment. Baseline clinical, virological, and vaginal microecological data were extracted from the electronic medical record system and laboratory databases. Follow-up HR-HPV test results at 12 months were obtained from the same institutional data source.

### Participants

**Figure 1:**
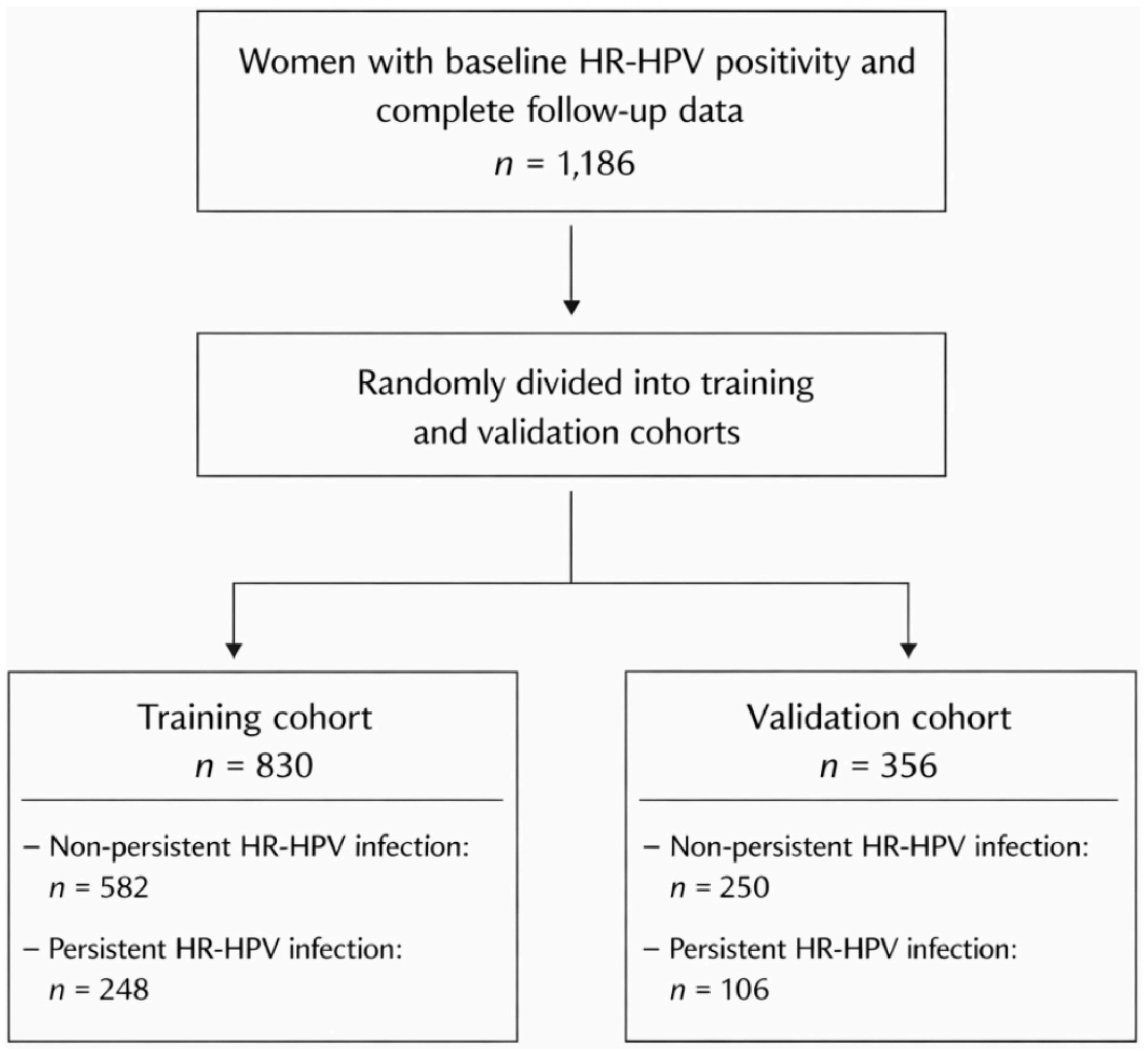
Flowchart of participant inclusion and cohort allocation in this retrospective study. A total of 1,186 women with baseline HR-HPV positivity and complete follow-up data were included. The study population was randomly divided into a training cohort (*n* = 830) and an internal validation cohort (*n* = 356). In the training cohort, 582 women tested negative for HR-HPV at follow-up and 248 had persistent/recurrent HR-HPV positivity at 12 months. In the internal validation cohort, 250 women tested negative for HR-HPV at follow-up and 106 had persistent/recurrent HR-HPV positivity at 12 months. HR-HPV, high-risk human papillomavirus.

Women were eligible for inclusion if they met all of the following criteria: (1) baseline positivity for HR-HPV; (2) available baseline vaginal microecological examination results; and (3) completed HR-HPV follow-up testing at 12 months. Women with incomplete baseline clinical data or missing follow-up HR-HPV results were excluded. A total of 1,186 women were finally included in the analysis. Among them, 832 tested negative for HR-HPV at follow-up and 354 were classified as having persistent/recurrent HR-HPV positivity at 12 months according to the operational outcome definition used in this study. The included participants were then randomly divided into a training cohort (*n* = 830) and an internal validation cohort (*n* = 356). The participant selection and cohort allocation process are shown in Figure 1.

### Variables

The primary outcome was persistent/recurrent HR-HPV positivity at 12 months, operationally defined as any HR-HPV positivity at follow-up among women who were HR-HPV-positive at baseline, regardless of whether the same HR-HPV genotype was detected at baseline and follow-up. Thus, women who tested positive for any HR-HPV genotype at 12 months were classified as having persistent/recurrent HR-HPV positivity, whereas women who tested negative for all HR-HPV genotypes at 12 months were classified as non-persistent. This outcome should not be interpreted as strictly type-specific HR-HPV persistence.

Candidate predictors were pre-specified according to clinical relevance and routine availability in gynecologic outpatient practice and included demographic characteristics, reproductive history, HPV genotype group, and vaginal microecological indicators. The variables considered in the initial analysis were age, body mass index (BMI), gravidity, parity, smoking status, HR-HPV genotype group, vaginal pH, vaginal cleanliness grade, Lactobacillus-dominant status, bacterial vaginosis, vulvovaginal candidiasis, Trichomonas vaginalis infection, and local inflammatory status. After penalized variable selection, seven predictors were retained for final model development: age, smoking status, HPV16/18 infection, vaginal pH, non-Lactobacillus-dominant microbiota, bacterial vaginosis, and moderate-to-severe local inflammation.

### Data sources

Baseline demographic and reproductive variables were obtained from the electronic medical record system. BMI was calculated as weight in kilograms divided by height in meters squared (kg/m^2^). Smoking status was recorded as a binary variable (yes/no). HR-HPV genotype group was categorized as HPV16/18 or other HR-HPV types according to baseline laboratory test results. Vaginal microecological variables were derived from routine vaginal discharge examinations performed by the hospital clinical laboratory, including vaginal pH measurement, wet mount microscopy, and Gram-stained smear evaluation. Briefly, vaginal discharge samples were collected from the lateral vaginal wall or posterior fornix using sterile swabs before any local treatment. Vaginal pH was measured using standardized pH indicator strips. Wet mount microscopy was used to assess vaginal cleanliness grade, leukocyte infiltration, Trichomonas vaginalis, and yeast-like fungal elements. Gram-stained smears were used to evaluate bacterial morphotypes, including Lactobacillus-like rods and mixed anaerobe-associated flora. No 16S rRNA next-generation sequencing or quantitative PCR assay was performed in this retrospective routine-care cohort. Vaginal pH was recorded as a continuous variable. Vaginal cleanliness grade was categorized as I–II or III–IV.

Lactobacillus status was classified as dominant or non-dominant according to microscopic assessment of Gram-stained vaginal smears and the original routine laboratory report. Lactobacillus-dominant microbiota was defined as Lactobacillus-like rods being reported as the pre-dominant vaginal flora, usually corresponding to moderate to numerous Lactobacillus-like rods on microscopic examination. Non-Lactobacillus-dominant microbiota was defined as absent or markedly reduced Lactobacillus-like rods, or the presence of mixed bacterial flora or anaerobe-associated morphotypes without Lactobacillus pre-dominance. Bacterial vaginosis, vulvovaginal candidiasis, and Trichomonas vaginalis infection were recorded as binary variables (yes/no) according to the routine laboratory report based on microscopy and microbiological assessment. Local inflammatory status was categorized as mild or moderate-to-severe according to the semi-quantitative leukocyte count and the original laboratory classification. Moderate-to-severe local inflammation was defined as increased leukocyte infiltration exceeding the mild category in the routine microscopic report. For regression modeling, vaginal pH was analyzed per 0.1-unit increase to improve clinical interpretability.

### Bias

Several measures were taken to reduce potential bias. First, only women with baseline HR-HPV positivity and complete follow-up data were included, thereby ensuring a clearly defined source population and outcome assessment. Second, all predictors were derived from routinely collected medical and laboratory records, which minimized recall bias. Third, predictor selection was based on clinical plausibility combined with penalized regression rather than on univariate significance testing alone, which reduced the risk of overfitting and unstable variable selection. Fourth, internal validation was performed using a randomly divided validation cohort to assess model reproducibility.

### Study size

The final study population comprised 1,186 women, including 354 cases of persistent/recurrent HR-HPV positivity. The training cohort included 830 women with 248 outcome events, whereas the internal validation cohort included 356 women with 106 outcome events. Given that seven predictors were retained in the final multivariable model, the number of events in the training cohort yielded an events-per-variable ratio well above the commonly recommended threshold of 10, supporting model stability.

### Quantitative variables

Continuous variables were summarized as mean ± standard deviation, and categorical variables were presented as number (percentage). For model development, age and vaginal pH were retained as continuous variables, whereas smoking status, HPV16/18 infection, non-Lactobacillus-dominant microbiota, bacterial vaginosis, and moderate-to-severe local inflammation were analyzed as binary variables. Vaginal pH was modeled per 0.1-unit increase to facilitate interpretation of the corresponding odds ratio.

### Statistical methods

All statistical analyses were performed using R software. Baseline characteristics were compared between women with and without persistent/recurrent HR-HPV positivity and between the training and internal validation cohorts. Continuous variables were compared using Welch's two-sample *t*-test, whereas categorical variables were compared using Pearson's chi-squared test. Candidate predictor selection was performed in the training cohort using least absolute shrinkage and selection operator (LASSO) regression with ten-fold cross-validation. Both the minimum lambda criterion (λ_min) and the one-standard-error criterion (λ_1se) were evaluated, and predictors retained at λ_1se were entered into the final multivariable logistic regression model to improve parsimony and stability. Univariate logistic regression was first used to examine the crude associations between the retained predictors and persistent/recurrent HR-HPV positivity. Multivariable logistic regression was then performed to estimate adjusted odds ratios (ORs) and 95% confidence intervals (CIs) for the final model. Multicollinearity among predictors in the final multivariable model was assessed using variance inflation factors (VIFs), with VIF values < 5 considered indicative of no obvious multicollinearity; the detailed results are presented in Supplementary Table 1. A nomogram was subsequently constructed on the basis of the final multivariable model.

Model discrimination was evaluated using the area under the receiver operating characteristic curve (AUC) with 95% CIs in both the training and internal validation cohorts. Calibration was assessed using calibration curves and the Hosmer–Lemeshow goodness-of-fit test. Clinical utility was evaluated by decision curve analysis (DCA). A two-sided *P*-value < 0.05 was considered statistically significant.

## Results

### Baseline characteristics of women with and without persistent/recurrent HR-HPV positivity

Table 1 summarizes the baseline characteristics of the 1,186 women included in the study, of whom 354 (29.8%) had persistent/recurrent HR-HPV positivity at 12 months and 832 (70.2%) tested negative for HR-HPV at follow-up. Women with persistent/recurrent HR-HPV positivity were significantly older than those who tested negative at follow-up (40.89 ± 8.45 vs. 37.87 ± 8.53 years, *P* < 0.001), whereas BMI was comparable between the two groups (22.95 ± 3.19 vs. 23.29 ± 3.13 kg/m^2^, *P* = 0.090). With respect to reproductive history, both gravidity and parity differed significantly between groups (*P* = 0.036 and *P* = 0.038, respectively). Smoking was more frequent among women with persistent/recurrent HR-HPV positivity than among those who tested negative at follow-up (14.4 vs. 5.6%, *P* < 0.001). In addition, HPV16/18 infection was more common in the persistent/recurrent HR-HPV positivity group (41.2 vs. 20.1%, *P* < 0.001). Vaginal microecological characteristics also differed substantially between the two groups. Women with persistent/recurrent HR-HPV positivity had a higher vaginal pH than those who tested negative at follow-up (4.85 ± 0.30 vs. 4.49 ± 0.34, *P* < 0.001). Poorer vaginal cleanliness (grade III–IV) was more prevalent in the persistent/recurrent HR-HPV positivity group (69.8 vs. 31.9%, *P* < 0.001), as was a non-dominant Lactobacillus status (81.1 vs. 35.1%, *P* < 0.001). Similarly, bacterial vaginosis (54.5 vs. 20.4%, *P* < 0.001), vulvovaginal candidiasis (18.4 vs. 10.9%, *P* < 0.001), and Trichomonas vaginalis infection (11.9 vs. 7.2%, *P* = 0.012) were all more common among women with persistent/recurrent HR-HPV positivity. Moderate-to-severe local inflammation was also observed more frequently in the persistent/recurrent HR-HPV positivity group than in those who tested negative at follow-up (57.1 vs. 30.0%, *P* < 0.001). Overall, persistent/recurrent HR-HPV positivity was associated with older age, smoking, HPV16/18 infection, and a disturbed vaginal microecological profile.

**Table 1:** Baseline characteristics of women with and without persistent/recurrent HR-HPV positivity.

Variable	Overall (*n* = 1,186)	HR-HPV negative at follow-up (*n* = 832)	Persistent/recurrent HR-HPV positivity (*n* = 354)	*P*-value
Age, years	38.77 ± 8.62	37.87 ± 8.53	40.89 ± 8.45	<0.001
BMI, kg/m^2^	23.19 ± 3.15	23.29 ± 3.13	22.95 ± 3.19	0.090
Gravidity				
0	56 (4.7)	47 (5.6)	9 (2.5)	0.036
1	146 (12.3)	113 (13.6)	33 (9.3)	
2	275 (23.2)	193 (23.2)	82 (23.2)	
3	307 (25.9)	216 (26.0)	91 (25.7)	
4	244 (20.6)	158 (19.0)	86 (24.3)	
5	123 (10.4)	83 (10.0)	40 (11.3)	
6	35 (3.0)	22 (2.6)	13 (3.7)	
Parity				
0	131 (11.0)	102 (12.3)	29 (8.2)	0.038
1	196 (16.5)	147 (17.7)	49 (13.8)	
2	279 (23.5)	196 (23.6)	83 (23.4)	
3	278 (23.4)	191 (23.0)	87 (24.6)	
4	302 (25.5)	196 (23.6)	106 (29.9)	
Smoking status, yes	98 (8.3)	47 (5.6)	51 (14.4)	<0.001
HR-HPV genotype group				
Other HR-HPV	873 (73.6)	665 (79.9)	208 (58.8)	<0.001
HPV16/18	313 (26.4)	167 (20.1)	146 (41.2)	
Vaginal pH	4.60 ± 0.36	4.49 ± 0.34	4.85 ± 0.30	<0.001
Vaginal cleanliness grade				
I–II	674 (56.8)	567 (68.1)	107 (30.2)	<0.001
III–IV	512 (43.2)	265 (31.9)	247 (69.8)	
Lactobacillus-dominant status				
Dominant	607 (51.2)	540 (64.9)	67 (18.9)	<0.001
Non-dominant	579 (48.8)	292 (35.1)	287 (81.1)	
Bacterial vaginosis, yes	363 (30.6)	170 (20.4)	193 (54.5)	<0.001
Vulvovaginal candidiasis, yes	156 (13.2)	91 (10.9)	65 (18.4)	<0.001
Trichomonas vaginalis infection, yes	102 (8.6)	60 (7.2)	42 (11.9)	0.012
Local inflammatory status				
Mild	734 (61.9)	582 (70.0)	152 (42.9)	<0.001
Moderate–severe	452 (38.1)	250 (30.0)	202 (57.1)	

Data are presented as mean ± standard deviation or n (%).

*P*-values were calculated using Welch's two-sample t-test for continuous variables and Pearson's chi-squared test for categorical variables.

BMI, body mass index; HR-HPV, high-risk human papillomavirus.

### Baseline characteristics of the training and internal validation cohorts

Table 2 presents the baseline characteristics of the training cohort (*n* = 830) and internal validation cohort (*n* = 356). Overall, the two cohorts were generally comparable with respect to most demographic, reproductive, virological, and vaginal microecological variables. Age (38.72 ± 8.69 vs. 38.88 ± 8.45 years, *P* = 0.767), BMI (23.16 ± 3.16 vs. 23.25 ± 3.14 kg/m^2^, *P* = 0.628), gravidity (*P* = 0.331), and parity (*P* = 0.186) did not differ significantly between the two cohorts. Similarly, the distribution of HR-HPV genotype group was identical in the training and validation cohorts (HPV16/18: 26.4% in both groups, *P* > 0.999). Vaginal pH (4.61 ± 0.36 vs. 4.58 ± 0.37, *P* = 0.214), vaginal cleanliness grade (*P* = 0.358), Lactobacillus-dominant status (*P* = 0.293), bacterial vaginosis (*P* = 0.193), vulvovaginal candidiasis (*P* = 0.804), Trichomonas vaginalis infection (*P* = 0.352), and local inflammatory status (*P* = 0.776) were also comparable between the two cohorts. Smoking status was the only variable that differed significantly between the two groups, with a higher proportion of smokers in the training cohort than in the validation cohort (9.4 vs. 5.6%, *P* = 0.040). Importantly, the incidence of persistent HR-HPV infection at 12 months was nearly identical between the training and internal validation cohorts (29.9 vs. 29.8%, *P* > 0.999), indicating an overall balanced distribution of outcome events after cohort allocation.

**Table 2:** Baseline characteristics of the training and internal validation cohorts.

Variable	Overall (*n* = 1,186)	Training cohort (*n* = 830)	Internal validation cohort (*n* = 356)	*P*-value
Age, years	38.77 ± 8.62	38.72 ± 8.69	38.88 ± 8.45	0.767
BMI, kg/m^2^	23.19 ± 3.15	23.16 ± 3.16	23.25 ± 3.14	0.628
Gravidity				
0	56 (4.7)	44 (5.3)	12 (3.4)	0.331
1	146 (12.3)	94 (11.3)	52 (14.6)	
2	275 (23.2)	184 (22.2)	91 (25.6)	
3	307 (25.9)	220 (26.5)	87 (24.4)	
4	244 (20.6)	173 (20.8)	71 (19.9)	
5	123 (10.4)	88 (10.6)	35 (9.8)	
6	35 (3.0)	27 (3.3)	8 (2.2)	
Parity				
0	131 (11.0)	97 (11.7)	34 (9.6)	0.186
1	196 (16.5)	124 (14.9)	72 (20.2)	
2	279 (23.5)	193 (23.3)	86 (24.2)	
3	278 (23.4)	200 (24.1)	78 (21.9)	
4	302 (25.5)	216 (26.0)	86 (24.2)	
Smoking status, yes	98 (8.3)	78 (9.4)	20 (5.6)	0.040
HR-HPV genotype group				
Other HR-HPV	873 (73.6)	611 (73.6)	262 (73.6)	>0.999
HPV16/18	313 (26.4)	219 (26.4)	94 (26.4)	
Vaginal pH	4.60 ± 0.36	4.61 ± 0.36	4.58 ± 0.37	0.214
Vaginal cleanliness grade				
I–II	674 (56.8)	464 (55.9)	210 (59.0)	0.358
III–IV	512 (43.2)	366 (44.1)	146 (41.0)	
Lactobacillus-dominant status				
Dominant	607 (51.2)	416 (50.1)	191 (53.7)	0.293
Non-dominant	579 (48.8)	414 (49.9)	165 (46.3)	
Bacterial vaginosis, yes	363 (30.6)	264 (31.8)	99 (27.8)	0.193
Vulvovaginal candidiasis, yes	156 (13.2)	111 (13.4)	45 (12.6)	0.804
Trichomonas vaginalis infection, yes	102 (8.6)	76 (9.2)	26 (7.3)	0.352
Local inflammatory status				
Mild	734 (61.9)	511 (61.6)	223 (62.6)	0.776
Moderate–severe	452 (38.1)	319 (38.4)	133 (37.4)	
Persistent/recurrent HR-HPV positivity at 12 months, yes	354 (29.8)	248 (29.9)	106 (29.8)	>0.999

Data are presented as mean ± standard deviation or n (%).

*P*-values were calculated using Welch's two-sample t-test for continuous variables and Pearson's chi-squared test for categorical variables.

BMI, body mass index; HR-HPV, high-risk human papillomavirus.

### Candidate predictor selection by LASSO regression in the training cohort

**Figure 2:**
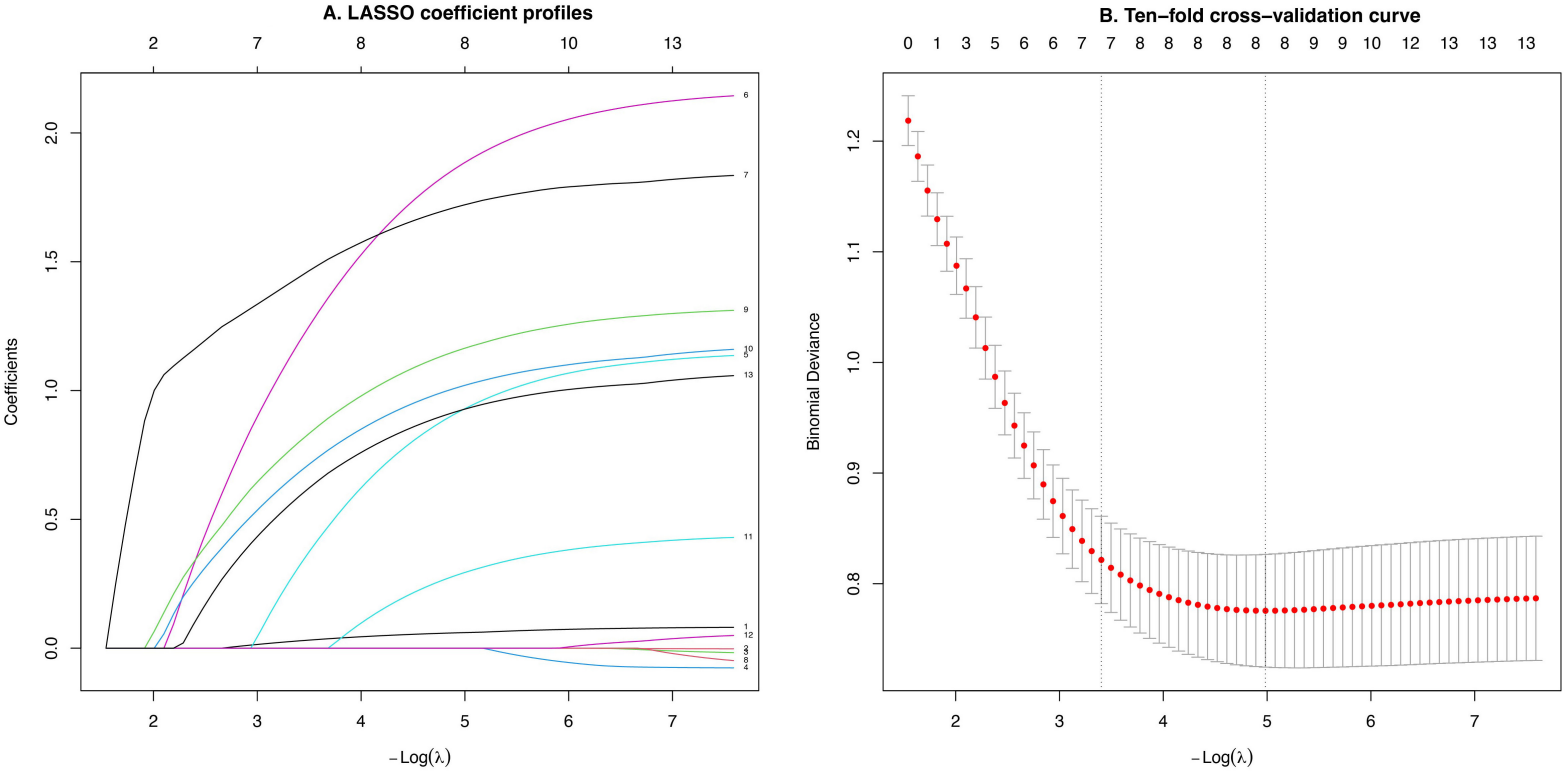
Candidate predictor selection using LASSO regression in the training cohort. Candidate predictor selection using least absolute shrinkage and selection operator (LASSO) regression in the training cohort. **(A)** LASSO coefficient profiles of the candidate predictors. Each colored curve represents the coefficient trajectory of an individual variable as the penalty parameter changes. **(B)** Ten-fold cross-validation curve for selection of the optimal penalty parameter (λ). The two dashed vertical lines indicate the values of λ at the minimum mean cross-validated error (λ_min) and at the one-standard-error criterion (λ_1se). Predictors retained at λ_1se were entered into the final multivariable logistic regression model for persistent/recurrent HR-HPV positivity. LASSO, least absolute shrinkage and selection operator.

Figure 2 and Table 3 present the results of candidate predictor selection using LASSO regression in the training cohort. As shown in the coefficient profile plot, regression coefficients gradually shrank toward zero with increasing penalization, whereas the ten-fold cross-validation curve was used to determine the optimal penalty parameter. Based on the one-standard-error criterion (λ1se), seven predictors were retained for subsequent model development. Specifically, the variables selected at λ1se were age, smoking status, HPV16/18 infection, vaginal pH, non-Lactobacillus-dominant microbiota, bacterial vaginosis, and moderate-to-severe local inflammation. In contrast, BMI, gravidity, parity, vaginal cleanliness grade, and Trichomonas vaginalis infection were not retained, while vulvovaginal candidiasis was selected at λmin but excluded at λ1se, suggesting a relatively weaker and less stable contribution to model performance. Overall, the LASSO procedure identified a parsimonious set of demographic, virological, and vaginal microecological predictors, which were subsequently entered into the final multivariable logistic regression model.

**Table 3:** Candidate predictor selection by LASSO regression in the training cohort.

Variable	Coefficient at λmin	Selected at λmin	Coefficient at λ1se	Selected at λ1se
Age, years	0.0603	Yes	0.0284	Yes
BMI, kg/m^2^	0.0000	No	0.0000	No
Gravidity	0.0000	No	0.0000	No
Parity	0.0000	No	0.0000	No
Smoking status	0.9265	Yes	0.3181	Yes
HPV16/18 infection	1.8816	Yes	1.1883	Yes
Vaginal pH	1.7196	Yes	1.4401	Yes
Vaginal cleanliness grade (III–IV)	0.0000	No	0.0000	No
Non-Lactobacillus-dominant microbiota	1.1625	Yes	0.8005	Yes
Bacterial vaginosis	1.0185	Yes	0.6830	Yes
Vulvovaginal candidiasis	0.2919	Yes	0.0000	No
Trichomonas vaginalis infection	0.0000	No	0.0000	No
Moderate-to-severe local inflammation	0.9263	Yes	0.5894	Yes

Least absolute shrinkage and selection operator (LASSO) regression was performed in the training cohort for candidate predictor selection.

Predictors retained at λ1se were entered into the final multivariable logistic regression model for persistent/recurrent HR-HPV positivity.

### Univariate and multivariable logistic regression analyses for persistent HR-HPV infection in the training cohort

Table 4 shows the associations between the predictors selected by LASSO regression and persistent/recurrent HR-HPV positivity in the training cohort. In univariate analysis, all seven retained variables were significantly associated with persistent/recurrent HR-HPV positivity (all *P* < 0.001), including older age, smoking, HPV16/18 infection, higher vaginal pH, non-Lactobacillus-dominant microbiota, bacterial vaginosis, and moderate-to-severe local inflammation. In the multivariable logistic regression model, all seven predictors remained independently associated with persistent/recurrent HR-HPV positivity. Specifically, older age was associated with higher odds of persistent/recurrent HR-HPV positivity (OR 1.075, 95% CI 1.049–1.103, *P* < 0.001). Smoking was also an independent predictor (OR 3.058, 95% CI 1.633–5.784, *P* < 0.001). Among virological variables, HPV16/18 infection showed the strongest association with persistent/recurrent HR-HPV positivity (OR 8.564, 95% CI 5.383–13.955, *P* < 0.001). Regarding vaginal microecological characteristics, each 0.1-unit increase in vaginal pH was associated with a 20.5% increase in the odds of persistent/recurrent HR-HPV positivity (OR 1.205, 95% CI 1.107–1.315, *P* < 0.001). Non-Lactobacillus-dominant microbiota (OR 3.750, 95% CI 2.140–6.665, *P* < 0.001), bacterial vaginosis (OR 3.171, 95% CI 2.030–4.991, *P* < 0.001), and moderate-to-severe local inflammation (OR 2.938, 95% CI 1.960–4.433, *P* < 0.001) were likewise independently associated with persistent/recurrent HR-HPV positivity. Overall, these findings indicate that both host clinical characteristics and vaginal microecological disturbances were independently associated with persistent/recurrent HR-HPV positivity.

**Table 4:** Univariate and multivariable logistic regression analyses for persistent/recurrent HR-HPV positivity in the training cohort.

Variable	Univariate OR (95% CI)	*P-*value	Multivariable OR (95% CI)	*P-*value
Age, years	1.040 (1.022–1.058)	<0.001	1.075 (1.049–1.103)	<0.001
Smoking status (yes vs. no)	2.917 (1.819–4.694)	<0.001	3.058 (1.633–5.784)	<0.001
HPV16/18 infection (yes vs. no)	3.459 (2.500–4.798)	<0.001	8.564 (5.383–13.955)	<0.001
Vaginal pH (per 0.1-unit increase)	1.415 (1.339–1.500)	<0.001	1.205 (1.107–1.315)	<0.001
Non-Lactobacillus-dominant microbiota (yes vs. no)	8.213 (5.739–11.975)	<0.001	3.750 (2.140–6.665)	<0.001
Bacterial vaginosis (yes vs. no)	5.628 (4.082–7.803)	<0.001	3.171 (2.030–4.991)	<0.001
Moderate-to-severe local inflammation (yes vs. no)	3.648 (2.679–4.988)	<0.001	2.938 (1.960–4.433)	<0.001

Multivariable logistic regression included the seven predictors retained at λ1se in LASSO regression for persistent/recurrent HR-HPV positivity.

### Nomogram construction and model performance in the training and internal validation cohorts

**Figure 3:**
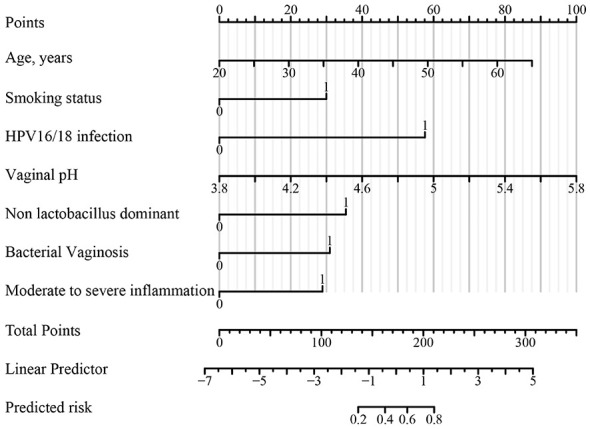
Nomogram for predicting persistent/recurrent HR-HPV positivity. Nomogram developed from the final multivariable logistic regression model for individualized prediction of persistent/recurrent HR-HPV positivity. The nomogram incorporates age, smoking status, HPV16/18 infection, vaginal pH, non-Lactobacillus-dominant microbiota, bacterial vaginosis, and moderate-to-severe local inflammation. For each predictor, the corresponding point value is assigned according to its position on the scale, and the total points correspond to the estimated probability of persistent/recurrent HR-HPV positivity. HR-HPV, high-risk human papillomavirus.

**Figure 4:**
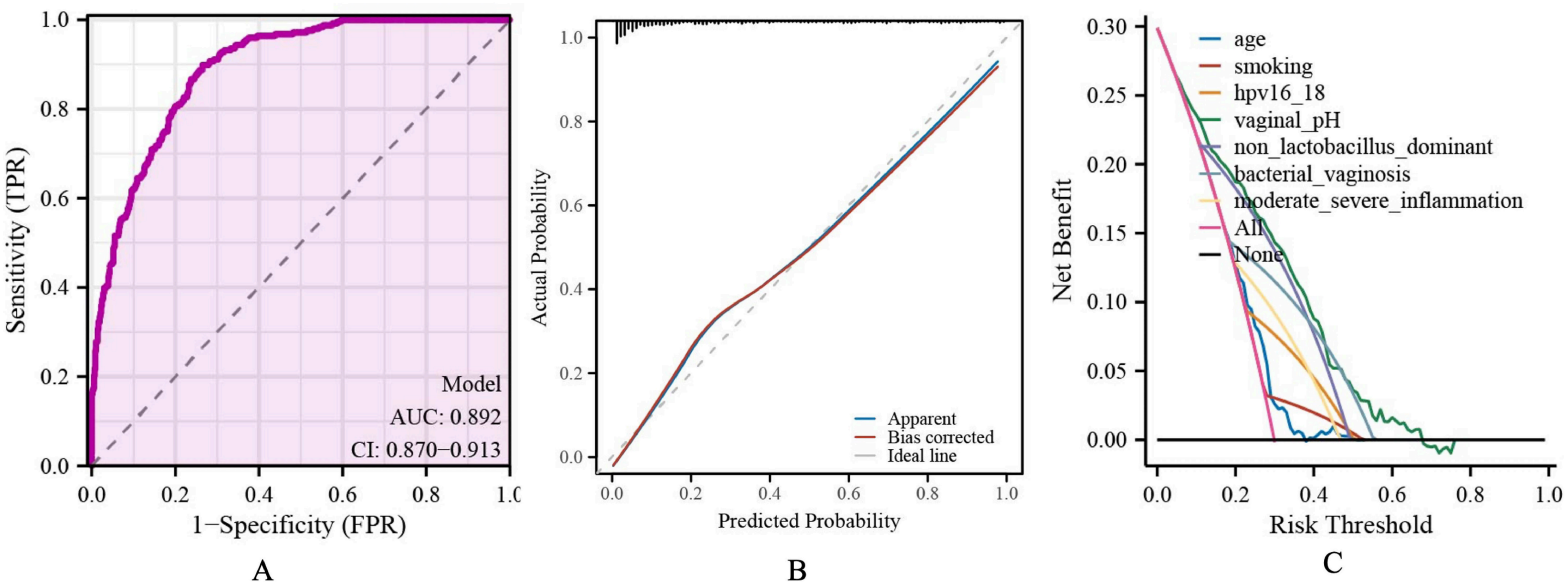
Performance of the prediction model in the training cohort. **(A)** Receiver operating characteristic (ROC) curve of the final model. **(B)** Calibration curve of the final model. **(C)** Decision curve analysis comparing the net benefit of the final model and individual predictors across a range of threshold probabilities in the training cohort.

**Figure 5:**
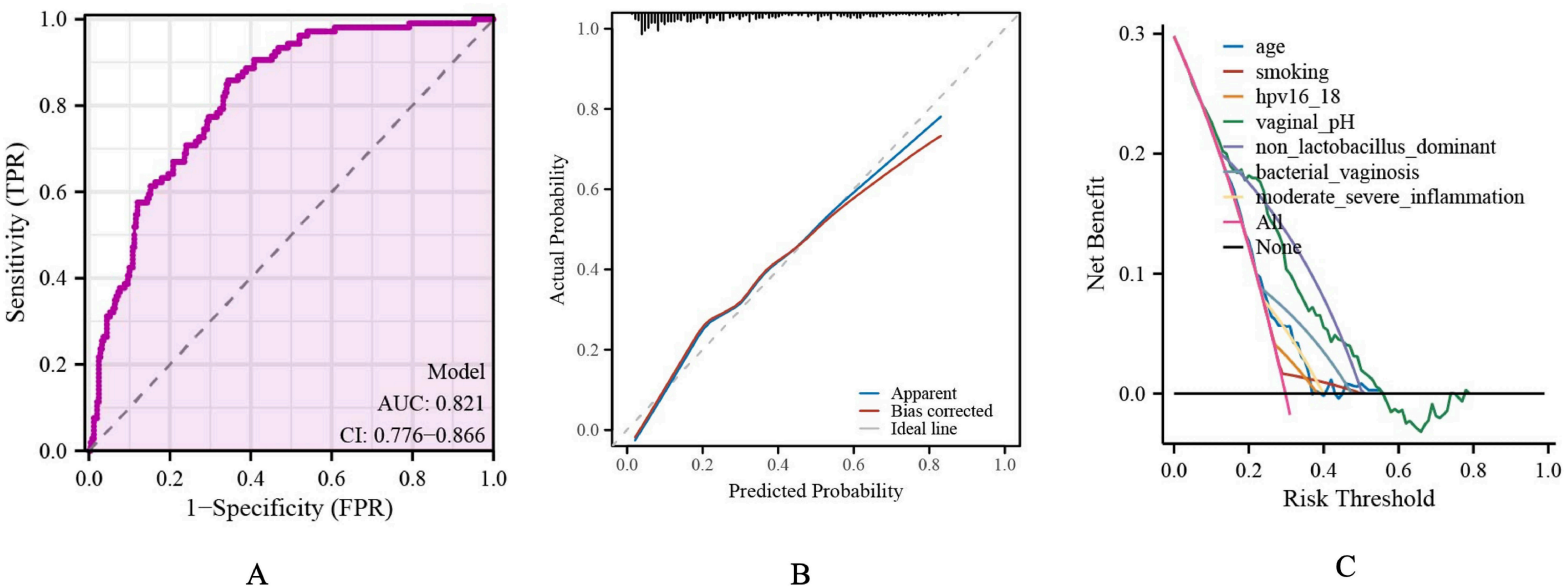
Performance of the prediction model in the internal validation cohort **(A)** Receiver operating characteristic (ROC) curve of the final model. **(B)** Calibration curve of the final model. **(C)** Decision curve analysis comparing the net benefit of the final model and individual predictors across a range of threshold probabilities in the internal validation cohort.

Based on the seven predictors retained in the final multivariable logistic regression model, a nomogram was developed to estimate the probability of persistent/recurrent HR-HPV positivity (Figure 3). The nomogram incorporated age, smoking status, HPV16/18 infection, vaginal pH, non-Lactobacillus-dominant microbiota, bacterial vaginosis, and moderate-to-severe local inflammation, allowing individualized risk estimation by summing the corresponding points for each predictor. The predictive performance of the model is summarized in Table 5 and illustrated in Figures 4 and 5. In the training cohort, the model showed good apparent discrimination, with an AUC of 0.892 (95% CI, 0.870–0.914). At the optimal cutoff, the sensitivity, specificity, and accuracy were 0.899, 0.735, and 0.784, respectively, with a positive predictive value of 0.592 and a negative predictive value of 0.945. The calibration curve demonstrated acceptable agreement between predicted and observed probabilities, and the Hosmer–Lemeshow test indicated adequate model fit (*P* = 0.149). Decision curve analysis further suggested potential net clinical benefit across a range of threshold probabilities (Figure 4). In the internal validation cohort, the model maintained acceptable discriminatory ability, with an AUC of 0.821 (95% CI, 0.775–0.867). The sensitivity, specificity, and accuracy were 0.858, 0.656, and 0.716, respectively, while the positive and negative predictive values were 0.514 and 0.916. Calibration in the internal validation cohort also appeared acceptable, supported by a Hosmer–Lemeshow *P*-value of 0.296. Decision curve analysis suggested potential clinical usefulness in the internal validation cohort (Figure 5). Overall, these findings suggest that the proposed model had promising discrimination, acceptable calibration, and potential clinical usefulness for identifying women with a higher probability of persistent/recurrent HR-HPV positivity within this internally validated cohort.

**Table 5:** Performance of the prediction model in the training and internal validation cohorts.

Metric	Training cohort	Internal validation cohort
Sample size	830	356
Number of events	248	106
AUC (95% CI)	0.892 (0.870–0.914)	0.821 (0.775–0.867)
Sensitivity	0.899	0.858
Specificity	0.735	0.656
Accuracy	0.784	0.716
Positive predictive value	0.592	0.514
Negative predictive value	0.945	0.916
Hosmer–Lemeshow *P-*value	0.149	0.296

Model discrimination was assessed using the area under the receiver operating characteristic curve (AUC), and calibration was assessed using the Hosmer–Lemeshow goodness-of-fit test in the training and internal validation cohorts.

## Discussion

In this retrospective observational study, we developed and internally validated a multivariable prediction model for persistent/recurrent high-risk human papillomavirus (HR-HPV) positivity that integrates clinical characteristics with vaginal microecological parameters. The final model showed promising discrimination and acceptable calibration in the training and internal validation cohorts, and decision curve analysis suggested potential clinical usefulness across a range of threshold probabilities.

Notably, the model incorporated seven routinely available predictors—age, smoking status, HPV16/18 infection, vaginal pH, non-Lactobacillus-dominant microbiota, bacterial vaginosis, and moderate-to-severe local inflammation—highlighting the predictive relevance of host factors, viral characteristics, and vaginal microecological status for persistent/recurrent HR-HPV positivity.

Our findings are consistent with prior evidence suggesting that persistent HR-HPV positivity is associated with multiple viral and host-related factors (16, 17). Among the predictors identified in this study, HPV16/18 infection showed the strongest association with persistence, which aligns with previous large cohort studies reporting that HPV16 and HPV18 confer a substantially higher risk of viral persistence and progression compared with other high-risk genotypes (18, 19). This observation may reflect intrinsic biological differences among HPV types, including variations in viral oncogene expression and immune evasion capacity (20).

Age was also independently associated with persistent/recurrent HR-HPV positivity, with older women showing a higher probability of persistent/recurrent positivity. This finding is consistent with prior studies suggesting that age-related changes in immune function, including immunosenescence and reduced mucosal immune responsiveness, may be related to reduced viral clearance (21, 22). In addition, smoking was identified as an independent predictor, which is in line with existing literature reporting associations between tobacco exposure, altered local immune responses, oxidative stress, and HPV persistence (23, 24).

Importantly, our study further highlights the predictive relevance of vaginal microecological status for persistent/recurrent HR-HPV positivity. A higher vaginal pH, non-Lactobacillus-dominant microbiota, bacterial vaginosis, and moderate-to-severe local inflammation were all independently associated with persistent/recurrent HR-HPV positivity. These findings support the growing body of evidence that vaginal dysbiosis is closely linked to HPV acquisition, persistence, and progression (25, 26). Lactobacillus species, particularly Lactobacillus crispatus, are known to be associated with a low vaginal pH, antimicrobial metabolite production, mucosal barrier integrity, and immune regulation (27). In contrast, Lactobacillus depletion and anaerobe-associated flora, as seen in bacterial vaginosis, have been associated with epithelial barrier disturbance, local inflammation, altered cytokine profiles, and reduced viral clearance (28).

The observed association between local inflammation and persistent/recurrent HR-HPV positivity is also biologically plausible. Chronic or excessive inflammation within the vaginal microenvironment has been reported to be associated with epithelial turnover, altered antigen presentation, and immune dysregulation, which may be relevant to persistent HPV positivity (29). Moreover, inflammation-related cytokines and immune mediators may be markers of a dysregulated local immune response associated with reduced HPV clearance (30).

From a clinical perspective, the internally validated prediction model developed in this study may have several potential applications after further external validation. First, it may provide a practical framework for individualized risk stratification using routinely available outpatient data, which may help identify women with a higher probability of persistent/recurrent HR-HPV positivity. Second, after external validation, such risk stratification may inform tailored follow-up strategies, including closer surveillance for individuals predicted to be at higher risk. Third, the inclusion of potentially modifiable factors, such as smoking and vaginal microecological abnormalities, may help clinicians identify areas for further assessment and counseling, although whether modifying these factors improves HR-HPV clearance requires prospective interventional studies.

This study has several strengths. The model was developed using a relatively large sample size with a sufficient number of outcome events, ensuring adequate statistical power and model stability. Predictor selection was performed using penalized regression (LASSO), which reduces the risk of overfitting and enhances model robustness. Furthermore, internal validation using a separate cohort provided an initial assessment of model performance. Importantly, all predictors included in the model are routinely available in clinical practice, supporting its feasibility for future external validation and potential clinical evaluation.

However, several limitations should be acknowledged. First, this was a single-center retrospective observational study, which may limit the generalizability of the findings to other populations or healthcare settings. Because of the observational design, the associations identified in this study should not be interpreted as causal relationships. External validation in independent cohorts is needed to confirm the model's transportability, calibration, and clinical applicability before broader implementation. Second, although we included a range of clinically relevant and microecological variables, other potential factors, such as host genetic susceptibility, detailed immune markers, sexual behavior, HPV vaccination status, and longitudinal changes in the vaginal microbiome, were not available in this dataset. In particular, HPV vaccination history was not systematically recorded in the electronic medical records or routine outpatient laboratory databases and therefore could not be reliably included in model development. The absence of HPV vaccination data may have introduced residual confounding and may affect the model's predictive accuracy and generalizability, especially when applied to populations with higher HPV vaccination coverage. In addition, the outcome in this retrospective study was defined as any HR-HPV positivity at 12 months among women who were HR-HPV-positive at baseline, rather than strict type-specific HR-HPV persistence. Therefore, women with clearance of the baseline genotype but positivity for a different HR-HPV genotype at follow-up may have been classified as having persistent/recurrent HR-HPV positivity. Future studies with paired genotype-specific HPV testing are needed to validate the model for strict type-specific HR-HPV persistence. Third, vaginal microecological assessment in routine clinical practice may have inherent variability, which could introduce measurement bias. Finally, although the model showed promising performance in this internally validated cohort, prospective studies are needed to evaluate its impact on clinical decision-making and patient outcomes.

In conclusion, we developed and internally validated a vaginal microecology-based multivariable prediction model for persistent/recurrent HR-HPV positivity. The model showed promising discrimination, acceptable calibration, and potential clinical usefulness within this single-center cohort. By integrating clinical and microecological predictors, this model may provide a practical approach for individualized risk assessment and may support risk-adapted follow-up planning in cervical cancer prevention after external validation. Future studies should focus on external validation and prospective evaluation to further establish its clinical applicability.

## Data Availability

The original contributions presented in the study are included in the article/Supplementary material, further inquiries can be directed to the corresponding author.
