# Supplementary material for: Development and internal validation of a vaginal microecology-based multivariable prediction model for persistent high-risk human papillomavirus infection: a retrospective study
Source: Front Med (Lausanne). 2026 Jul 3;13:1851813. doi: 10.3389/fmed.2026.1851813 (PMC13376309; doi:10.3389/fmed.2026.1851813)
Supplement: Supplementary file 1 [file Table_1.docx]

**Supplementary Table 1.** Variance inflation factor for multicollinearity assessment in the multivariable model

| **Variable** | **VIF** | **Interpretation** |
| --- | --- | --- |
| Age, years | 1.005 | No obvious multicollinearity |
| Smoking status | 1.017 | No obvious multicollinearity |
| HPV16/18 infection | 1.008 | No obvious multicollinearity |
| Vaginal pH (per 0.1-unit increase) | 2.412 | No obvious multicollinearity |
| Non-Lactobacillus-dominant microbiota | 2.015 | No obvious multicollinearity |
| Bacterial vaginosis | 1.409 | No obvious multicollinearity |
| Moderate-to-severe local inflammation | 1.102 | No obvious multicollinearity |

**Note:** Variance inflation factor (VIF) values <5 were considered to indicate no obvious multicollinearity among predictors included in the final multivariable logistic regression model.
